# Mr. Cross's Sketches of the Medical Schools of Paris

**Published:** 1816-03

**Authors:** 


### *Mr. Cross's Sketches of the Medical Schools of Paris.*

( Continued from p. 148. )

In continuing the review of Mr. Cross's publication, upon the plan prescribed to ourselves in the last Number, we shall endeavour to delineate an outline of the remarks made by that gentleman during his residence in Paris; which, general and rapid as it must necessarily be, may enable the British reader to form a tolerably correct notion of the actual state of surgical practice in the French metropolitan hospitals. It is, in our opinion, much to be regretted, that Mr. Cross did not considerably prolong his visit to those fertile and interesting scenes of professional observation; and that his views were almost exclusively directed to the treatment of surgical diseases. We mean not, however, to insinuate, that, on the score of positive information, the practitioner of this island will sustain much loss by the omission ; for a detail of the antiquated pathology and inert and temporizing practice of the Parisian physician would serve only to alloy with a less manly and honourable feeling that exultation in the superiority of British surgery, which perusal of these Sketches must necessarily excite: yet it is scarcely possible that the mind can trace, upon the chart of an intelligent and unprejudiced observer, like Mr. Cross, the progress made in an important branch of science by a great and rival people, from personal intercourse with whom we have been so long shut out, and not feel deeply interested, and even indirectly instructed, by the retrospect. To the heart of nations, as of individuals, contemplation of error and imperfection in those around is calculated to convey a lesson quite as salutary as the spectacle of purest excellence, and often more permanently impressed. The intellectual improvements of a country, it may perhaps be said, will be sufficiently appreciated by reference to her literature. This observation, to a certain degree correct, must not be admitted without some limitations. The sciences, like the forms and opinions, and propensities of mankind, have in general their national peculiarities. The minuter shades of these, which, although trifling in themselves, give, when blended, a strong colouring to the picture, will rarely be imparted even by the more delicate and lucid descriptions of a native. The reason is obvious: no one can delineate things or qualities, of the existence of which he is inconscious. Hence, an observant and enlightened foreigner will communicate, by a few strokes of his pen, more correct notions of the progress and peculiar character of French medicine than may be gleaned, on this side of the channel, in twelve months, from all the costly cargoes imported by the London booksellers. This truth, with respect to surgery, the animated Sketches of Mr. Cross serve most completely to illustrate.

We shall concentrate the substance of Mr. Cross's observations under the following heads: *Hernia, Hospital Gangrene, Fractures, Injuries of the Head, Stricture of the Urethra, Aneurism, Diseased Testis, Cancer, Syphilis, Cutaneous Affections, Infantile Diseases.* Such scattered fragments as this arrangement will not comprehend we shall afterwards collect, to serve as the materials of a *concluding Section.*

*Hernia.* — In speaking of M. Dupuytren's lectures on operative surgery, we observed, that he demonstrated the anatomy of hernia without any reference to the fascial coverings. A singular occurrence, which he had sometimes witnessed after the operation for hernia, was mentioned in one of these lectures. It took place in corpulent subjects, where the omentum, overloaded with fat and adhering strongly to the sac, had been allowed to remain externally, after the intestine was returned.

"The portion of epiploon, thus left, has undergone a kind of decomposition (without inflammation, without gangrene), and oily globules have constantly distilled from it: an oily fluid, of a rancid odour, has continued to be furnished by the over fat epiploon within the abdomen, and been discharged through the wound made at the time of the operation. The patient has sunk, gotten into low fever and died, from the long continuance of this oily discharge."

Dupuytren, therefore, recommends that the omentum, whenever thus loaded with fat, should be detached from its adhesions and returned; an operation far less to be dreaded than accession of the morbid state which we have just mentioned, and which, it is thought by Mr. Cross, may be induced by the after-treatment of the wound; union by the first intention being commonly prevented by the French surgeons. Of two cases of operation for strangulated hernia which came under his notice, we shall detail one, as very clearly exhibiting the various steps of the process executed according to the French mode, and other peculiarities of practice and opinion, calculated rather to astonish than instruct the British reader. The patient, a female aged 80, was admitted into *L'Hotel Dieu*, at one o'clock, with crural hernia, nine days strangulated. During this period, no stool had passed. The vomiting, which had existed from the beginning, recurred only, for the last twenty-four hours, on taking aliment. The tumor was small, and almost painless on pressure; the abdomen much distended and tense: the pulse 80, and feeble from advanced age. At six o'clock, when Dupuytren arrived, no attempt had been made to return the intestine, or palliate the symptoms. The surgeon inferred, from a hasty and superficial examination, that mortification had taken place; pronounced the case desperate; and, "without disturbing the position of the patient or trying to reduce the tumor," proceeded directly to the operation.

"Dupuytren began by pinching up the skin transversely, puncturing it with the bistoury, and cutting upwards. The first incision thus made, was enlarged at both ends, and another was made that crossed it in the middle, at right angles. The operation was pursued, by dissecting back the four angles of skin. He scratched a hole in the successive layers, covering the tumor, and enlarged it with the scissars, still cutting the parts in correspondence with the crucial incision of the integuments. He was very cautious in these steps of the operation, remarking to us, as he went on, that from the time the strangulation had existed, there might be great adhesion of the intestine to the fore part of the sac. At length a small puncture was made into the cavity of the sac, as was shewn by the escape of half an ounce of fluid. The sac was then laid open by cutting in four directions from this point (still corresponding to the first crucial incision), and the intestine very unexpectedly presented a favourable appearance, being of a dull red colour. Blood oozed freely through a slight scratch that had been accidentally made upon its surface. Anterior to the intestine, there was a small portion of omentum, which adhered firmly to the sac. A part of the lower and upper portion of the intestine, within the abdomen, was pulled down, 'to see if it was in a good state.' The operator then put his fore-finger as far as the stricture would allow, and passing a probe-pointed bistoury beyond the end of his finger, he cut the stricture slightly, in a direction 'upwards and a little outwards.' The intestine was readily reduced, but the small piece of omentum was still left in the sac, to which it was adherent. No attention was paid to closing the wound afterwards; and although, from the great laxity and abundance of the skin, the cut edges almost fell into apposition, no care was taken to keep them so. A thin piece of linen, spread with cerate, and having numerous small holes cut in it, was placed upon the wound: a compress and roller completed the dressing. The patient was ordered a half pint emollient injection every four hours, and a little wine and water at the same time, on account of her weakness and great age. Shortly after the first injection, there was a copious evacuation, and it was not necessary to repeat it."

The case terminated most favourably. The operation for crural hernia was performed on another female, after the employment of the warm bath and slight attempts at reduction. The wound was not dressed again till the fifth morning after. Five weeks passed ere it had completely healed. In general, the French adopt the excellent practice of operating early for hernia. Venæsection is employed where the pain and pulse indicate it: nothing was previously attempted where the intestine had been long strangulated. Union of the wound by the first intention is rarely encouraged. Carbon and brandy were applied, in one case, to the sore attacked with hospital gangrene sixteen days after the operation, and the sloughing was arrested.

*Hospital Gangrene.*—This dreadful malady frequently makes its appearance in the French hospitals. No reliance is placed, by the surgeons, upon internal remedies in their treatment of it, although acknowledged to be a constitutional and contagious affection. The topical application of vegetable and diluted mineral acids proves successful in mild cases; but actual cautery alone is found capable of arresting the fatal progress of the more unfavourable. "The iron is applied red hot, so as to procure an eschar on every point of the surface of the sore."

*Fractures.*—Great attention is paid to the treatment of simple fractures in the French hospitals. The application of splints and bandages is deferred until the tension and inflammation have been subdued by warm fomentations and poultices. The splints, in general clumsy, are very carefully padded. Frequently, linen bags filled with oat chaff are used for this purpose. The bandages are nicely applied, and the apparatus is never suffered to become loose from neglect. In fractures of the lower extremities, the patient is commonly laid on his back with the limb extended. Dupuytren sometimes prefers a slightly bent position. Respecting fracture of the neck of the femur, Mr. Cross enters into a somewhat long discussion. It is the general opinion of British surgeons, that *osseous* reunion never takes place when the neck of the thigh-bone is fractured *completely within* the orbicular ligament. The French think differently. But Mr. Cross saw nothing in their dissections or morbid preparations, that could alter his view of this controverted point. Indeed, it appears that the French do not clearly understand the premises upon which the argument rests with us; for they produced in refutation specimens of re-united bone, where the fracture had not been confined to the portion within the capsule, but extended obliquely from the neck to the body of the femur. These were obviously inadequate to decide the question. Dr. Macartney, of Dublin, possesses a preparation in which a double fracture of the femur is exhibited.

"One fracture is external to the capsule, separating the neck of the bone and the great trochanter; and union has taken place by a sort of ligamento-cartilaginous substance. The other is a fracture of the neck within the capsule: here no union has taken place at all, and the greater part of the separated head of the bone has been absorbed."

This certainly is weighty evidence in favour of our insular disputants; yet the final solution of this problem in pathology can only be determined by rigorous and reiterated examinations of fractured femur. The treatment of *fractured patella,* Mr. Cross mentions in terms of merited commendation. The reader will recollect, that the principal object in this case is, to place, and preserve, the great extensor muscles of the leg in the utmost possible state of relaxation. This, as the *rectus femoris* has an attachment to the pelvic bones, will not be sufficiently accomplished by extending the leg upon the thigh; but the thigh also must be bent upon the trunk. In the instance witnessed by Mr. Cross, this position seems to have been rigorously observed by the French surgeons. No bandage was used until the tension and inflammation of the injured part had subsided; and, after ten days, the apparatus was re-applied. Previously to removal of the bandages, the patient was carefully moved into another bed; and the surgeon began by rolling the limb upwards from the foot, and downwards from the groin, nearly to the joint. The extremity of each of the two rollers was left unemployed.

"Then a piece of linen, divided into three strips at one end, was fastened by the additional part of the roller above the knee, and a similar piece of linen was also fastened by the roller below the knee. Compresses, dipt in warm lotion, were laid on each side the patella. The treble ends of the two pieces of linen were intermixed with each other, and pulled in opposite directions so as to approximate, or rather to keep approximated, the two portions of the patella. These pieces of linen being pulled sufficiently tight, were fastened by additional rollers round the leg and round the thigh. A pasteboard splint was put under the knee and calf of the leg; and bags, filled with oat-chaff, were put on each side of the limb, and tied by several tapes."

A guard was placed over the foot, as in other fractures. On the whole, great attention is paid by the French to the position of the limb in fractures—an important part of surgical practice too frequently neglected in this island.

*Injuries of the Head.*—The operation of the *trepan* is frequently had recourse to. The proscriptions of the illustrious Dessault, respecting it, have lost the influence which they must once have exerted on the practice of the French surgeons. Indeed, these gentlemen seem to manœuvre on the crania of their patients with less diffidence and circumspection than the British operator would feel himself called upon to exercise. In one or two of the cases, observed by Mr. Cross, wherein the trepan was applied, there existed " no symptoms to urge the immediate performance of the operation." Dupuytren operated with success on the right parietal bone of a female, which had been denuded twenty days before, but shewed neither fracture nor depression: a small quantity of pus, lodged between the cranium and dura mater, was evacuated. All the urgent symptoms, which first came on at the end of fifteen days—shiverings, head-ache, tinnitus aurium, and vomiting, were permanently relieved. The patient had been repeatedly bled soon after the occurrence of the accident. A fourth case terminated fatally: Dubois raised the depressed portion of the frontal bone, while the subject, a female, was yet suffering from the effects of the concussion. Sensibility was not impaired: the system displayed no "evident symptoms of compression." Lint and common cerate were employed to dress the wound. On the 17th day from the operation, the patient, previously going on well, was seized with vomiting, fever, and low delirium. She died on the 20th.

"Fifty hours after death, the head was inspected in the public theatre, before the students left the hospital. The fracture of the frontal bone had extended into the orbit: a spicula of bone had pierced the *dura mater*, and was still remaining, imbedded in the brain; there was pus between the hemispheres of the brain, and diffused from the injured part as far back as the cerebellum; and a portion of the right anterior lobe of the cerebrum had a gangrenous appearance."

Few British surgeons, of the present day, would, we believe, have declined operating under these circumstances. The *trephine* is very rarely employed.

*Urethral Stricture.* The caustic bougie is denounced by the French surgeons, as a "very dangerous and harsh remedy," without any experience in its employment. We, however, agree with Mr. Cross, that treatment of stricture by the *conical silver catheter** is not very remarkable for its mildness. Our readers will, probably, be of the same opinion, when they have gone through the case recorded by Mr. Cross, to illustrate the application of the instrument. A middle-aged man suffered from permanent stricture. He had dysuria, but not complete retention. Gentle attempts to pass an instrument into the bladder failed. M. Roux, with a conical catheter, slightly curved and almost pointed at the extremity, forced a way into the bladder. The pressure on the instrument was regularly applied, and the direction of its point determined by observing the lateral rings. The depression of the outer or distal extremity of the instrument was begun, when the finger of the operator in the rectum felt that its point had reached

* An engraving of this instrument is given. The curvature of it is very small.

the "apex of the prostate." The operation was very painful. Immediate escape of the urine was prevented by plugging the orifice of the catheter, which was depressed between the thighs and there secured, to prevent the inner extremity of the instrument from being drawn out of the bladder, as must necessarily have happened, had its outer end been raised to the abdomen. The catheter is commonly retained in the urethra three or four days; but, in this case, the sufferings of the patient were such, that it was removed after twenty-four hours, and a small elastic gum catheter without difficulty substituted; its extremity properly secured and plugged, so as to allow the evacuation of the urine only at certain periods. Great relief was the consequence. On the fourth day, the testicle, scrotum, and perinæum were swollen. Poultices were applied; the catheter continued. The swelling subsided, and a larger gum catheter was introduced after four days. In a fortnight more, the instrument was again changed, and replaced every six or eight days by one of a still larger size. At the end of six weeks, the urethra was capable of receiving a full-sized catheter. It is asserted, that unfavourable consequences rarely result from this treatment; but Roux, in his clinical lecture, mentioned two cases of fatal termination, where the conical catheter had been employed. In the first, a false passage had been made, and was followed by infiltration of urine, inflammation, and sloughing. Peritoneal inflammation was induced in the second, from the instrument passing between the pubis and bladder. Mr. Cross accuses the French surgeons of employing this formidable treatment with too little discrimination as to the nature of the obstruction, which is to be overcome. He justly observes, that it is only admissible in cases of old cartilaginous stricture, especially when complicated with *fistula in perinæo:* and here, all experienced surgeons occasionally employ means very nearly resembling "the *forcing* method of the French." The conical catheter has, in one instance, been used by Mr. Astley Cooper, with perfect success. Dubois sometimes employs a mild treatment in stricture of the urethra. A small bougie is passed up to the obstruction, and left pressing against it: the urine commonly escapes by the side of the instrument. In a few days, it admits of being passed farther, till, at last, it enters the bladder. A small gum catheter is now substituted, and replaced, at intervals, by one of a larger size, until the largest can be introduced. These instruments may be permanently worn in the urethra with very little inconvenience. The incrustation, which forms upon the inner extremity, is the only circumstance which demands their removal, except when the gradual increase of size is an object with the surgeon. The period in which this deposition takes place, may vary from seven or eight days to as many weeks; and the progress of it may be known by the corresponding diminution of the stream of urine: while that remains unimpaired, the catheter needs not be removed. The French surgeons leave the orifice of the instrument unclosed in cases of *fistula in perinæo* combined with stricture; but in simple stricture, paralysis of the bladder, and other diseases of these organs, the urine is only allowed to escape four or five times a-day. For an account of the French mode of manufacturing gum catheters, we refer the reader to our miscellaneous department.

( To be concluded in our next. )

