# Erratum

**Published:** 1816-04

**Authors:** 


*Erratum.*—P. 360, l. 23, *for* few British, *read* most British.

